# The development and evolution of arthropod tagmata

**DOI:** 10.1098/rspb.2024.2950

**Published:** 2025-04-16

**Authors:** Ariel D. Chipman

**Affiliations:** ^1^ Department of Ecology, Evolution & Behavior, The Silberman Institute of Life Science, The Hebrew University of Jerusalem, Jerusalem, Israel

**Keywords:** arthropods, tagma, segment identity, Hox genes, body plan

## Abstract

The segmented body is a hallmark of the arthropod body plan. Morphological segments are formed during embryogenesis, through a complex procedure involving the activation of a series of gene regulatory networks. The segments of the arthropod body are organized into functional units known as tagmata, and these tagmata are different among the arthropod classes (e.g. head, thorax and abdomen in insects). Based on embryological work on segment generation in a number of arthropod species, coupled with a survey of classical descriptions of arthropod development, I suggest a new framework for the evolution of arthropod tagmata. The ancestral condition involves three developmental tagmata: the pre-gnathal segments, a tagma that is formed within a pre-existing developmental field and a tagma that is formed through the activity of a segment-addition zone that may be embryonic or post-embryonic. These embryonic tagmata may fuse post-embryonically to generate more complex adult tagmata. This framework is consistent with the evolution of tagmosis seen in the early arthropod fossil record. It also calls for a re-thinking of the decades-old division of arthropod development into short-germ versus long-germ development, a re-thinking of questions of segment identity determination and the role of Hox genes in tagma differentiation.

## 1. Overview

The arthropod body plan is characterized by repeated morphological units, or segments, along the antero–posterior axis. These segments are usually organized into higher level units, each composed of several segments, known as tagmata (tagma in singular). The segments of each tagma normally have shared functions, and roughly similar size and shape, and they are often differentiated from the segments of the adjoining tagmata by a distinct morphological boundary. While the nature and composition of the tagmata vary among arthropod classes, they are more or less conserved at the level of the class, and are often used as defining characters of the class. The term ‘tagma’ itself is used relatively loosely in the literature, corresponding to the inherent variability in tagmata. The morphological literature has given a lot of attention to the defining features of different tagmata, and to the variability in their composition, but there has been almost no recent discussion of the evolutionary history of the tagmata or of their development. I present a synthesis of what is known about tagmosis (the arrangement of segments into tagmata) from comparative morphology, from developmental biology and from the fossil record. I use this synthesis to suggest a novel model for the evolution of arthropod tagmata. I argue that conserved elements of developmental tagmosis form the basis for much of the observable tagmata.

The details of arthropod tagmosis have been extensively reviewed by Fusco & Minelli [1], and will not be repeated here. I will give a brief overview of the typical tagmosis pattern in the main arthropod lineages, emphasizing the most likely plesiomorphic condition for each lineage.

### (a) Chelicerates

The basic chelicerate body plan is composed of two tagmata, usually known as the prosoma (or cephalothorax) and opisthosoma (or abdomen). This is most clearly seen in the terrestrial arachnids, exemplified by spiders. The prosoma includes four pairs of walking appendages and anterior to them two pairs of feeding or sensory appendages, the pedipalps and the chelicerae. The anterior-most segment of the prosoma carries the eyes and the labrum. The opisthosoma normally does not carry walking appendages, but may carry breathing appendages, or specialized structures such as spinnerets.

There are numerous minor variations on this theme, and these have been reviewed extensively by Dunlop & Lamsdell [2]. In pycnogonids, the number of walking appendages can vary, and the opisthosoma is rudimentary or nonexistent [3]. Some arachnids have a subdivided opisthosoma (e.g. scorpions), whereas in others there is no clear border between the prosoma and opisthosoma (e.g. acarids, opiliones). Nonetheless, it is generally accepted that the two-tagma structure is ancestral to extant chelicerates, as are four pairs of walking appendages and two sensory/feeding appendage pairs in the prosoma [2].

### (b) Myriapods

The myriapod body plan consists of a head and a mostly homonomous trunk. The head is composed of six segments: three pre-gnathal segments and three gnathal segments. This is the composition of the head in all members of Mandibulata (Myriapoda + Pancrustacea). The pre-gnathal segments include the three parts of the brain and carry eyes on the first segment and a pair of antennae on the third segment. The gnathal segments carry feeding appendages [4].

The trunk is composed of all of the remaining segments, most of which carry a pair of walking appendages. The myriapod trunk may display a number of lineage specific novelties, such as a mid-body transition zone in many centipedes, or a mismatch between dorsal and ventral segmentation in millipedes. There may be specialized segments, such as the centipede venom-claw or the posterior-most segment, or gonopods used for sperm transfer in millipedes. In some cases, there is some degree of regionalization within the trunk [4]. Nonetheless, none of these specializations alters the basic tagmosis of the myriapod body plan, which is always composed of a head and a trunk.

### (c) Pancrustaceans

The non-hexapod pancrustaceans display a bewildering array of tagmosis patterns [5]. In all cases, the anterior-most tagma is a six-segment mandibulate head, although the head is not always separated by a clear morphological boundary from the tagma behind it. Indeed, the head is often covered by a head shield or carapace that also covers segments posterior to the head. The region covered by the carapace is sometimes known as a cephalothorax, although this is a very different structure from the one known by the same name in chelicerates.

The trunk of malacostracan crustaceans is usually divided into two regions, known as a pereon and pleon. The pereon carries walking appendages, as well as maxillipedes, which are modified appendages used in feeding. The pleon usually does not have walking appendages, but often has other modified appendages. In brachyurans, as well as in other ‘crab-like’ crustaceans, most or all of the pleon is reduced and folded ventrally under the pereon [5,6].

### (d) Hexapods

Insects and their close relatives in Hexapoda have the most consistent pattern of tagmosis among arthropods. The body is composed of three sharply defined tagmata: a six-segment mandibulate head, a three-segment thorax with walking appendages and an abdomen with usually between 9 and 11 segments or as few as 6 in Collembola, which normally does not carry appendages [7].

There are minor variations on this structure. For example, in some hymenopterans, the border between the thorax and the abdomen shifts. The head and thorax are sometimes fused, as in some psyllids. In some holometabolous insects, there is a reduction in the number of adult abdominal segments. Nonetheless, there is little doubt that the ancestral pattern of tagmosis is the common three-tagma structure.

## 2. The fossil record

The earliest arthropod stem-group members, the paraphyletic assemblage known as lobopodians, show no tagmosis, and are thus similar to the extant arthropod sister groups, onychophorans and tardigrades [8]. The head is composed of a single segment, with no additional segments attached to it [9]. While there may be some regionalization and specialization of specific segments along the trunk, these are not grouped into functional regions and do not display morphological borders, and should thus not be considered as tagmata.

The gilled lobopodians (such as *Kerygmachela* and *Pambdelurion*), as well as diverse organisms as *Opabinia* and the radiodonts, also have a single-segment head, and no obvious tagmata within the trunk. The single head carries the eyes and usually a single pair of large raptorial or sensory appendages [10,11]. Members of these groups display varying degrees of regionalization. The size of the segments and appendages varies along the body axis, but without a sharp discontinuity. The neck region in radiodonts is a distinct group of 4−5 segments, but it is not delimited by a morphological boundary. The tail fan is a distinct structure in some gilled lobopodians and radiodonts, and it may be composed of a number of segments [12,13]. All this considered, while members of the gilled lobopodian/radiodont grade start to show increasing levels of regionalization, there are no obviously differentiated tagmata.

Deuteropoda is the clade that includes both crown-group arthropods and the upper stem group. Members of Deuteropoda have three pre-gnathal segments [14]. In the upper stem group, these three segments comprise the entire head (but see O’Flynn *et al*. [15] for a different viewpoint). These head segments often bear specialized appendages (sometimes called ‘great appendages’). In some cases, there are 1−3 specialized appendages just posterior to the head [16–19]. It is possible to describe these animals as having a distinct head and trunk, as in myriapods, although the head is composed of only three segments. It is not clear whether the specialized post-cephalic appendages should be considered part of an expanded head, as in extant mandibulates, or modified trunk segments, since there is no sharp morphological boundary. These appendages may be the precursors of the gnathal segments in mandibulate arthropods.

Tagmosis in trilobites and its development have been studied fairly extensively due to the excellent fossil record of these animals, which allows the reconstruction of complete post-embryonic developmental series, most notably in *Aulacopleura konincki* [20,21]. The trilobite body is divided into three tagmata: the cephalon (or head), thorax and pygidium. The posterior border of the cephalon is clear and stable and is established in the earliest recoverable stages, which have a head only, or a head with a single additional segment. However, the thorax and pygidium are dynamic, with new segments being added to the pygidium in successive moults, and then ‘released’ to the thorax, to maintain a more-or-less stable number of pygidial segments, and a growing number of thoracic segments [22]. The thorax–pygidium border is thus a dynamic border, and the tagmata themselves vary in number of segments and in the border between them throughout ontogeny. In this sense, they are unlike the tagmata in all extant arthropods and in most fossil arthropods.

Stem-group chelicerates all have a distinction between a prosoma and an opisthosoma, but the number of segments in each of these tagmata and the identity of the appendages carried on these segments do not always conform to what is found in extant chelicerates. The diversity and evolution of tagmata in fossil chelicerate taxa were also discussed in the review by Dunlop & Lamsdell [2]. Stem-group mandibulates have a typical head tagma and variable posterior tagmata. Their diversity has been reviewed by Waloszek & Maas [23]. A more recent alternative framework for the mandibulate stem group is outlined by Izquierdo-López & Caron [24]. They reconstruct only two segments posterior to the pre-gnathal segments, rather than the three found in extant mandibulates.

## 3. Developmental tagmosis

The basis for tagma differentiation is laid down during embryonic development. While this statement sounds intuitively almost obvious, the embryonic basis of tagmosis has hardly been studied, and has only been looked at explicitly in a handful of species. There is, however, enough implicit information in both classical and modern descriptions of embryonic development to be able to draw some general conclusions. I argue that embryonic regionalization of segments forms a more conserved and ancient tagmosis than the apparent tagmosis seen in the adult body plan.

The connection between regionalization of embryonic segments and adult tagmata is not direct, and there is often a mismatch between the two. The most obvious example is in the PGS. There is an ongoing debate about the evolutionary origin of these units [25,26], with Lev & Chipman [27] arguing that they should not even be considered segments. However, regardless of these differing opinions, there is no doubt that the three anterior segments in all arthropods develop differently and stand on their own as a distinct embryonic unit, despite being incorporated into the adult head (in mandibulates) or prosoma (in chelicerates).

In mandibulates, the three posterior segments of the head, the gnathal segments, are indistinguishable from the segments immediately posterior to them during the early stages of development. In most insects, the gnathal and thoracic segments (abbreviated as gnatho-thoracic segments hereafter) form a distinct series of six segments that form together and are of similar size and shape, until the differentiation of the appendages (mouthparts or walking legs) [28–31]. In myriapods, the gnathal segments are identical to trunk segments in the early germ-band before appendage differentiation [32,33]. In chelicerates, prosomal segments and opisthosomal segments have different morphologies [34–36], although this is probably mostly due to the presence of walking limbs on prosomal segments, which affect the segments’ morphology.

In many cases, there are also differences in the way different segments are formed. In what follows, I review what is known about these differences in species where all segments are formed during embryogenesis (epimorphic development). Species where some of the segments are formed post-embryonically (anamorphic development) are discussed in §4.

### (a) Insects

**Figure 1.**
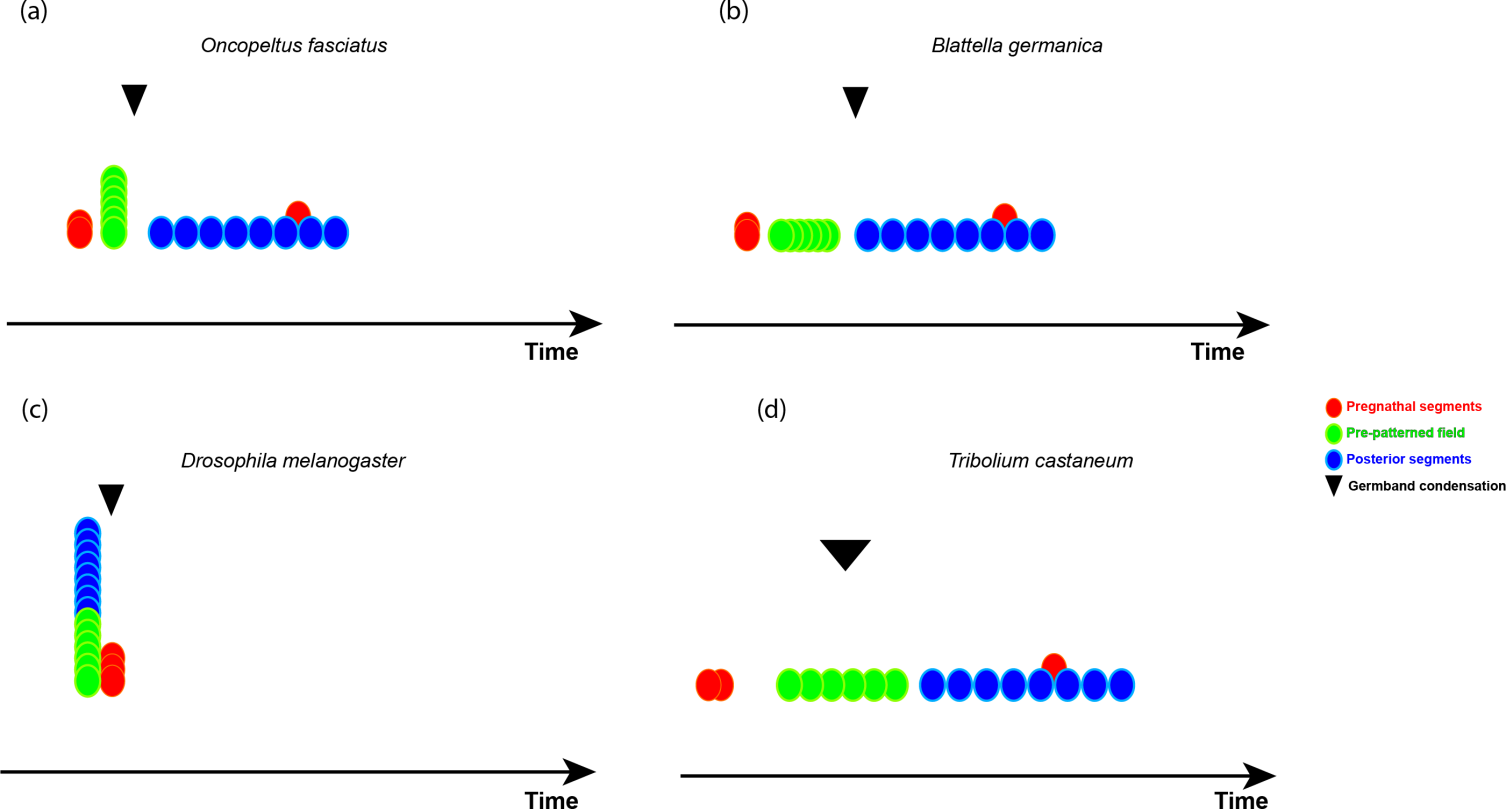
Schematic representation of the different modes of segment generation in four insect species. (a) In the milkweed bug *Oncopeltus fasciatus*, two of the pre-gnathal segments (red) and the gnatho-thoracic segments (green) are patterned nearly simultaneously in the blastoderm. Germ-band condensation (marked with a black arrowhead) occurs through a process of invagination. The abdominal segments (blue) are patterned sequentially in the germ band. The third pre-gnathal segment, the intercalary segment, is patterned during abdominal segmentation, as in many insects. (b) In the German cockroach *Blattella germanica,* there is no sharp distinction between a blastoderm and germ-band stage. However, the gnatho-thoracic segments are patterned rapidly and sequentially in a pre-patterned field, which then condenses to form the germ-band, where abdominal segmentation takes place. (c) In the well-studied fruit fly, *Drosophila melanogaster*, all segments are patterned simultaneously in the blastoderm, followed rapidly by the condensation of the germ-band. (d) In the flour beetle *Tribolium castaneum*, all segments are patterned sequentially. However, there is a difference in rate between the gnatho-thoracic segments and the abdominal segments, and the segment-addition zone is only active during abdominal segmentation. Germ-band condensation occurs simultaneously with gnatho-thoracic segmentation (marked with a broad black arrowhead).

The similarity between gnathal and thoracic segments is not limited to their morphology in the germ-band stage. In many cases, they also develop through a similar developmental programme. This similarity has been best demonstrated in the milkweed bug *Oncopeltus fasciatus* (figure 1a). In this species (and in other hemipterans), early development can be divided into a blastoderm stage and a germ-band stage [37–39]. In the blastoderm stage, the embryo is composed of a single layer of cells covering an ovoid yolk mass. The cells of the blastoderm then undergo a process of invagination, to form a germ-band that is embedded inside the yolk. The segmentation process begins already in the blastoderm stage, with orthologues of the *Drosophila* segmentation cascade genes being expressed in sequence to form segmental stripes of segment polarity genes such as *engrailed* and *wingless* [40]. During this early phase of segmentation, the gnatho-thoracic segments are patterned at the molecular level, and the borders between them are established by the interactions of the segment-polarity network. There is no morphological evidence of segmental borders during the blastoderm stage.

During the invagination process, the embryonic tissues condense to give the germ-band, and shortly afterwards, the gnatho-thoracic segments can be seen morphologically [41]. About halfway through the invagination process, a specialized embryonic zone forms at the posterior of the germ band, and it is from this posterior zone, known as the segment addition zone (SAZ) or growth zone, that the abdominal segments start appearing sequentially [42].

The question arises whether this distinction between gnatho-thoracic and abdominal segmentation is unique to hemipterans, or whether a similar distinction can be seen in the development of other insects. An analysis of an additional species of hemimetabolous insects, together with a survey of reported segmentation patterns in other insects, suggests that this may be a general pattern—at least in hemimetabolous insects—although the details may vary. In the German cockroach *Blattella germanica* (figure 1a), the gnathal and thoracic segments are patterned sequentially at the level of segment polarity genes, but the pair rule gene *even-skipped* is expressed more or less simultaneously in all gnatho-thoracic segments, and fades sequentially, with *hedgehog* expression coming up in its place. Only after all of the gnatho-thoracic *hedgehog* expression stripes come up, does the SAZ form and abdominal segments appear sequentially, driven by a cyclic expression of *even-skipped* [31].

In the cricket *Gryllus bimaculatus,* gnatho-thoracic stripes of *hedgehog* expression come up rapidly and sequentially. There is then a gap of a few hours before the SAZ forms and abdominal segments arise sequentially [43]. This pattern is similar to that seen in *B. germanica*.

A closer analysis of the pattern in these three hemimetabolous insects shows that the main difference between the formation of gnatho-thoracic and abdominal segments is not whether they are formed simultaneously or sequentially. The main difference is in the embryonic environment in which they are formed. Gnatho-thoracic segments are patterned within a pre-existing embryonic field or anlage, whereas abdominal segments are formed from a posterior domain where axial elongation is taking place—the SAZ.

Detailed descriptions of segmentation in additional hemimetabolous insects are rare, but a survey of the literature suggests a similar pattern in many cases (see electronic supplementary material, table S1 for examples). Anterior segments form within a pre-existing field, without addition of new tissue, and posterior segments form from an SAZ. The border between the two processes is not always evident from the existing description, but the data are consistent with the border being the thoracic-abdominal border. Even in short-germ insects such as the grasshopper *Schistocerca*, the early embryonic field encompasses only the gnathal segments and expands through tissue condensation to include the thoracic segments [28]. Only after this process is done are abdominal segments added sequentially.

Since hemimetabolous insects form a paraphyletic group, basal to the more widely studied Holometabola, we can assume that the pattern described above is the ancestral segmentation mode for insects. This pattern was thus presumably lost in Holometabola, otherwise, it would have been discovered before, given the extensive body of work on holometabolan development (e.g. *Drosophila melanogaster*; figure 1c). But is this really the case? In the well-studied red flour beetle *Tribolium castaneum* (figure 1d), a study of the dynamics of segment generation showed that the thoracic segments form at a uniform rate, there is then a slowing down of the process, followed by a dramatic increase in segmentation rate during abdominal segmentation [44]. Indeed, looking at the germ band of *T. castaneum*, the SAZ is only evident during abdominal segmentation, with gnatho-thoracic segments being patterned in a rapidly condensing—but not extending—embryonic field.

In the parasitic jewel wasp *Nasonia vitripennis*, where there is no obvious SAZ, there is a transition in segment generation between the six anterior segments (the gnatho-thoracic segments) and the posterior abdominal segments [45]. The first three pair-rule stripes (corresponding to the six anterior segments) arise simultaneously and the following stripes arise in what has been called ‘progressive segmentation’—e.g. sequential segmentation without an SAZ [46].

Even in *Drosophila melanogaster,* the paradigm for simultaneous, long-germ segmentation, there are two patterning centres: an anterior patterning centre responsible for gnathal and thoracic segments, and a posterior centre responsible for abdominal segments [47–49]. Even when all obvious evidence of a difference in segmentation mode between segments has been lost, there is a vestige hinting at an ancestral distinction between two groups of segments.

### (b) Crustaceans

The diverse morphologies and tagmosis modes seen in crustaceans are reflected in diverse modes of segmentation. There is not a lot of information about embryonic segmentation in different crustaceans. Most crustaceans for which we have any information display indirect development, hatching as a larva with only three visible segments—two pairs of antennal segments and the mandibular segment. This type of larva is known as a nauplius [50], and is discussed in §4 on post-embryonic segmentation.

Species with direct development are found throughout crustacean diversity. In many cases, even when all segments are formed during embryogenesis, there is an early developmental stage where the three naupliar segments develop and start differentiating, before the appearance of any other segments. This stage is called the ‘egg nauplius’, and is usually understood to be homologous to the recently hatched nauplius of indirect developing species [51,52]. An example of such development can be seen in the branchiopod *Daphnia magna* [53,54]. In this species, the naupliar segments appear very early in development, followed by all posterior segments. However, the posterior segments do not follow a strict anterior–posterior sequence. The two maxillary segments, which will form part of the adult head, appear at a slight delay relative to the thoracic segments, suggesting the possibility of a somewhat different mechanism underlying their formation, relative to the sequential, SAZ-based formation of thoracic segments.

The crustacean group in which embryonic development has been best studied is Malacostraca. Most malacostracan crustaceans form their segments through posterior stem cells known as ectoteloblasts [55,56]. In this mode of segmentation, every division of the ectoteloblasts generates a daughter cell that will be the precursor to cells of a single parasegment. Segmentation via ectoteloblasts has been studied in the isopod *Porcellio scaber* (among others) [57–59]. The anterior segments, including the pre-gnathal and gnathal segments, are formed through cell rearrangements prior to the activity of the ectoteloblasts. All segments from the first thoracic segment onwards are formed sequentially via divisions of the ectoteloblast row. It is worth noting that in post-hatching morphology, the cephalothorax, which is the anterior-most tagma, includes the pre-gnathal and gnathal segments, in addition to the first thoracic segment.

Studying the neural development of two crayfish species (members of Malacostraca), Vilpoux *et al*. [60] showed that the central nervous system in the naupliar region (pre-gnathal + mandibular neuromeres) develops almost simultaneously. After its formation, there is a short lag, after which the posterior neuromeres develop sequentially.

The best studied model for crustacean embryonic segmentation is the amphipod *Parhyale hawaiensis*. However, similar to *D. melanogaster*, and in common with all amphipods, *P. hawaiensis* displays a simultaneous mode of segment generation, which is almost certainly derived [61,62]. In all amphipods, ectodermal cells assemble into a grid, without going through the sequential ectoteloblast divisions typical of other malacostracans. The pre-gnathal and mandibular segments (the naupliar segments) display a slightly different mode of assembly, whereas there is no noticeable difference in the way all other segments are formed.

### (c) Myriapods

**Figure 2.**
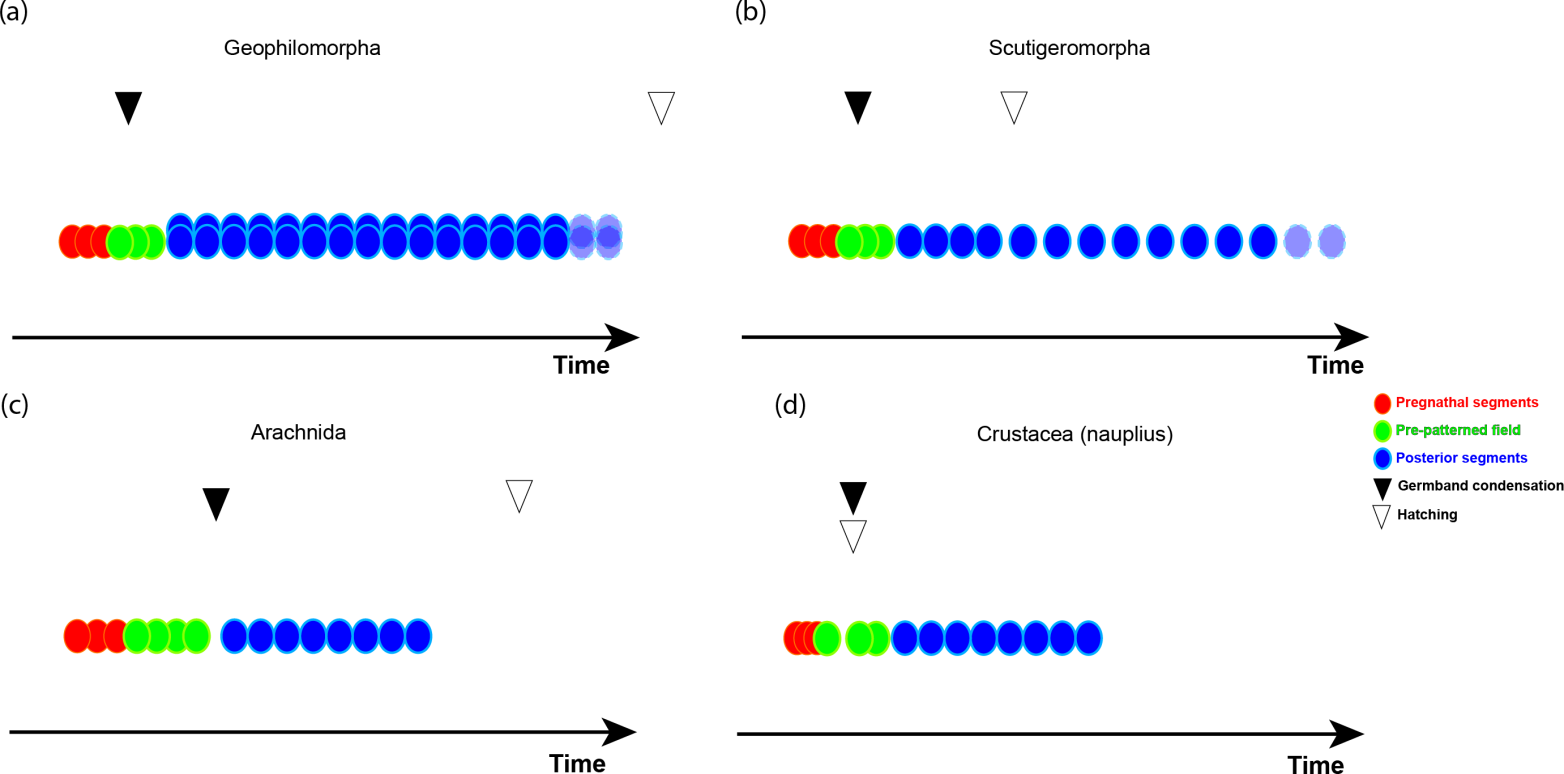
Schematic representation of the different modes of segmentation in four arthropod species. (a) In geophilomorph centipedes (such as *Strigamia maritima*), the germ band condenses (black arrowhead) during the segmentation of the pre-gnathal segments (red) and the gnathal segments (green), without the activity of a segment addition zone. Trunk segments (blue) are formed mostly two at a time (transparent fill and dotted lines indicate that not all segments are portrayed). Segmentation ends well before hatching (white arrowhead). (b) In scutigeromorph centipedes, anterior segmentation is probably similar to that of geophilomorph centipedes, although there is very little data). Four trunk segments form during embryogenesis, with successive segments added post-hatching. (c) In arachnids, including spiders and others, the pre-gnathal segments and the limb-bearing segments are patterned rapidly within the early embryonic disc. The disc then condenses to give the germ-band, and opisthosomal segments are patterned sequentially from a segment-addition zone. (d) In crustaceans that have a nauplius stage, the pre-gnathal segments and the mandibular segments are patterned embryonically. The germ-band condenses and the nauplius hatches, with additional segments added sequentially. In crustaceans without a nauplius stage (not shown), the main difference is that hatching is heterochronically shifted to after the end of segmentation.

In the centipede *Strigamia maritima* (figure 2a), a ‘head bulge’ appears from the germ disc before segmentation begins, and the PGS form within this bulge [33]. The gnathal segments, the segment bearing the venom-claw and the first leg-bearing segment appear nearly simultaneously [32,33,63]. All posterior segments form sequentially, initially with a two-segment periodicity of a segmentation clock [64]. There is no apparent difference in the segmentation of the gnathal and trunk segments. Towards the end of the segmentation process, there is a shift from a pattern where two segments are patterned from each stripe of *even-skipped* expression to one where each *even-skipped* stripe generates a single segment [65].

The only diplopod species where segmentation has been studied is the pill millipede *Glomeris marginata* [66]. Segmentation in this species is not very different from that of the centipede *S. maritima*. Segmentation is sequential throughout, with the first few segments appearing more or less simultaneously, with no obvious difference between gnathal segments and trunk segments. Typically for diplopods, there is a mismatch between dorsal and ventral segmental patterning, with each dorsal tergite corresponding to two ventral leg-bearing segments. This mismatch is only seen in the trunk segments, and not in the head segments, where dorsal and ventral segments are aligned. The anterior-most trunk segment also shows no dorsoventral mismatch [66].

### (d) Chelicerates

In spider segmentation, the prosomal segments are patterned rapidly within the initial germ disc, without any extension of the disc (figure 2c). After prosomal segments are formed, the SAZ begins to function, generating single segments sequentially through a cyclic process [35,67–69]. This pattern is seen both in the wandering spider *Cupiennius salei* and in the house spider *Parasteatoda tepidariorum*.

A similar pattern is seen in other arachnids where segmentation has been studied, although the details are usually not as clear. Both in the whip scorpion *Phrynus marginemacultus* [70] and in the harvestman *Phalangium opilio* [71], the prosomal segments are formed rapidly within the germ disc, while the opisthosomal segments are formed sequentially from an SAZ.

## 4. Post-embryonic segmentation

There are two main modes of post-embryonic (or anamorphic) segmentation found within arthropods. The first is addition of segments within a nauplius/post-nauplius larva (figure 2d). In this mode, segment addition takes place in a free-swimming larva that hatches with a minimal number of segments. Segment addition is not normally accompanied by moulting. Although there are moults during the segment addition process, they are few relative to the number of segments added. The process occurs continuously and at a relatively constant rate [72]. This mode of post-embryonic segmentation is found in some crustacean taxa and may be ancestral to Pancrustacea.

The second mode is the addition of segments in sequential moults, in an animal that hatches with functional walking limbs. This is the case in a number of myriapod taxa and in some crustaceans, and it is also found to a limited extent (addition of a single post-embryonic segment) in some mites and in oniscidian isopods.

Post-naupliar segment addition has been studied in only a handful of crustacean species [72–74]. In all cases, the two antennal segments and the mandibular segment form rapidly in embryogenesis. The development of the ocular segment, which lies anterior to the antennal segments, is usually not described explicitly, but it is probably determined early in larval development. The post-mandibular segments arise sequentially, and there is no apparent difference in their mode or dynamics of development. In the fairy shrimp *Thamnocephalus platyurus*, the thoracic and abdominal segments are different in shape and size, but arise similarly [72].

Centipedes of the orders Scutigeromorpha (figure 2b) and Lithobiomorpha hatch from the egg with four or six to eight leg-bearing segments, respectively. Additional segments are added one or two at a time in successive moults, up to a total of 15 leg-bearing segments [4]. Most millipedes also hatch with seven leg-bearing segments and add segments in successive moults, with no clear final number of segments [4].

The development of the pycnogonids (sea spiders) is very diverse, but the most common mode of development involves the hatching of a pronymphon larva that includes the pre-gnathal segments (hereafter PGS) and an additional one or two segments, similar to the situation in crustaceans with a nauplius. Additional segments are added in successive moults [75].

The arthropod fossil record indicates that post-embryonic segment addition was much more phylogenetically diverse in the past. A number of fossil taxa have a rich enough fossil record that allows reconstruction of post-embryonic developmental series. This has already been discussed above for trilobites, and it is important to point out here that the earliest developmental stage found for trilobites is a head larva, with no externally obvious segmentation. It is not clear how many segments these larvae contain, but based on exceptionally preserved fossils of adult trilobite heads, they probably contain five segments, presumably including the PGS and two additional cephalic appendages [76].

‘Orsten’-type fossilization is an exceptional mode of preservation wherein small individuals are fossilized in exquisite detail through phosphatic replacement of organic tissues. Late Cambrian Orsten fossils provide a wealth of information about larval development in crustaceans and their relatives. These fossils indicate that the nauplius is a very early invention within the crustacean lineage, and that sequential post-naupliar segment addition was established and common by the late Cambrian [77,78].

Developmental series for a number of stem and putative crown group arthropods have been published in the past decade. These include the radiodont *Stanleycaris* [79,80], the upper stem arthropods *Isoxys* [81] and *Chandianella* [82], and the putative crown arthropods *Leanchoilia* [83,84] and *Fuxianhuia* [85]. All of these cases indicate that post-embryonic segment addition was found throughout arthropod phylogeny in the early stages of their evolution. However, none of these developmental series is as complete as that of trilobites, so we do not know the minimal number of segments with which these animals hatched from the egg.

## 5. A model for the evolution of arthropod tagmosis

**Figure 3.**
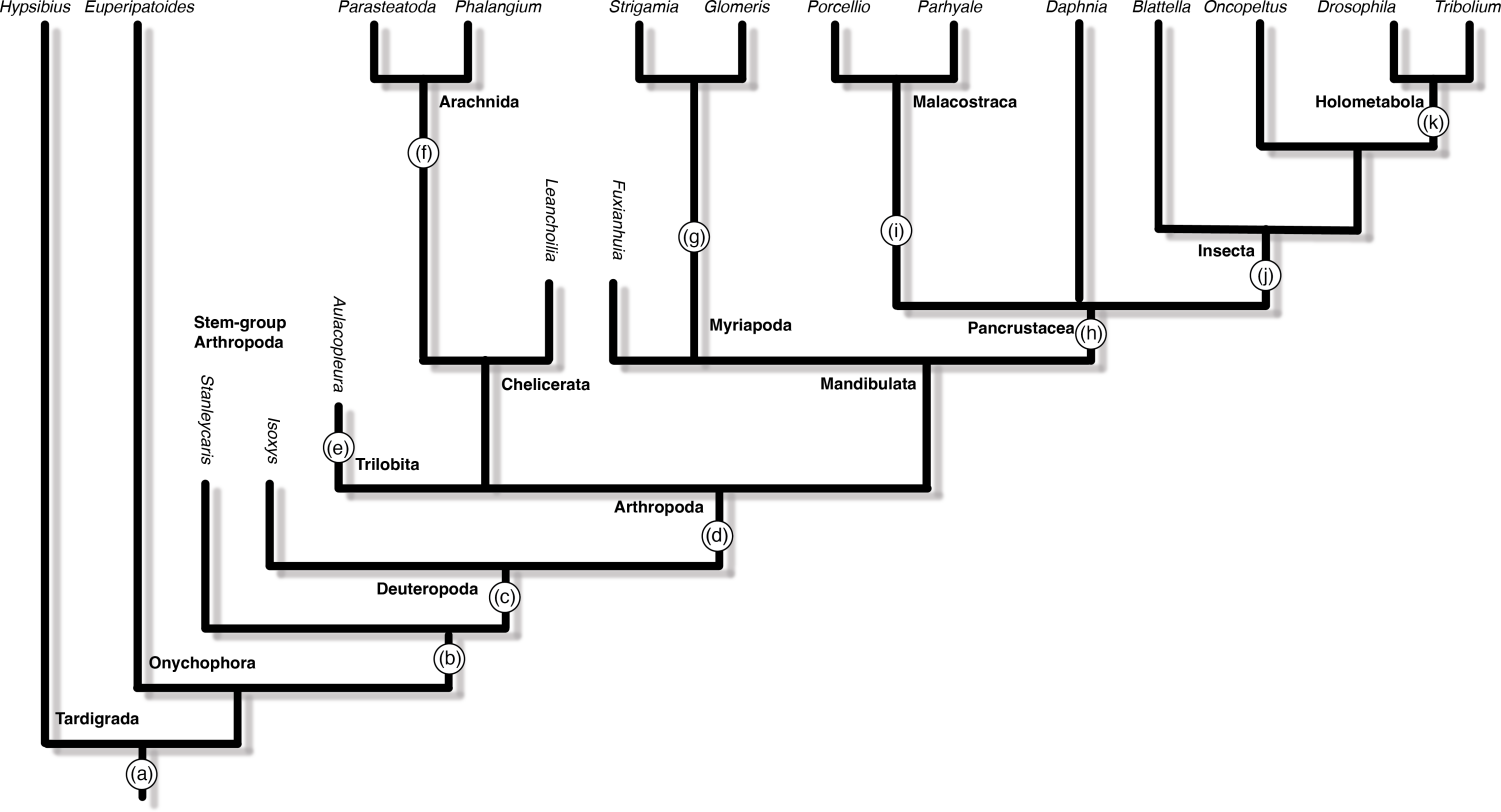
A schematic tree of the main panarthropod groups mentioned in the text, with the major evolutionary events related to tagmosis mapped on the tree. The tips are extant genera for which there exist developmental data. Genera listed vertically represent fossil species for which we have ontogenetic data. (a) In the common ancestor of Panarthropoda, there was a single-segment head and an undifferentiated trunk region. (b) Post-embryonic segment addition appeared in stem-group arthropods. This may also represent the first appearance of a segment addition zone. (c) Deuteropoda is characterized by the appearance of the three-segment head, representing a novel developmental tagma with a unique mode of segment generation: the pre-gnathal region. (d) The common ancestor of Arthropoda already had three distinct developmental tagmata: the pre-gnathal segments, a tagma including segments developing in a pre-existing field, and a tagma with segments generated from a segment addition zone. (e) Trilobites have a unique mode of tagmosis, involving segment release between the pygidium and the thorax. (f) All extant arachnids have a prosoma composed of the pre-gnathal segments and an embryonic tagma with four segments formed in a pre-existing field, and an opisthosoma, with a variable number of segments formed from a segment addition zone. In stem arachnids, the number of segments formed in each tagma varies, but the general arrangement is the same as in extant arachnids. (g) In myriapods, the number of segments formed within a pre-existing field is three or four, including the gnathal segments and possibly one post-gnathal segment. (h) The nauplius appeared early in the evolution of Pancrustacea, although it is not clear if it is a synapomorphy of the entire clade. All pancrustaceans have a pre-gnathal region and a tagma including segments generated from a growth zone. The number of segments developing within a pre-existing field is variable and may be as low as a single segment in some lineages. (i) In Malacostraca, the segment addition zone functions via specialized stem cells: ectoteloblasts. (j) In insects, the thorax, a novel tagma, first appears. It is composed of three of the segments formed within a pre-existing field, with an additional three segments, the gnathal segments, fusing with the head as in other mandibulates. (k) In Holometabola, the developmental distinction between gnatho-thoracic segments and abdominal segments is masked, with the evolution of novel segmentation modes.

The many different patterns of tagmosis found in extant arthropods emerged from an unknown ancestral pattern. I suggest that ancestral tagmosis was originally a developmental phenomenon, with the segments of diverse regions of the animal patterned differently. Over evolutionary time, the segments that were patterned using different developmental mechanisms evolved differential morphologies and different functional roles. The original differences in development are preserved to varying extents in extant arthropods. In many cases, the borders between regions that are patterned differently are preserved as tagma borders. In some cases, the mode of development has changed so the developmental borders are not immediately obvious. In many cases, additional tagmata evolved as sub-divisions of the original ones. In other cases, there has been fusion within and between adult tagmata, masking the original borders, although these can still be seen in development (figure 3).

In the common ancestor of all arthropods, segments were patterned via three mechanisms: (i) the anterior three segments (the PGS) were patterned separately and not always in sequence with other segments, via a developmental mechanism that did not involve pair-rule gene homologues, and probably including a mechanism of ‘stripe-splitting’; (ii) a number of trunk segments, probably numbering on the order of 5−10 segments, were patterned within a pre-existing embryonic field, either by subdivision of the field (simultaneous segmentation) or through a sequential process that did not involve the addition of new embryonic tissue (progressive segmentation); and (iii) posterior segments were patterned sequentially, through the activity of a segment addition zone (growth zone), which involved axis extension via a combination of the generation of new tissue through cell division and of convergent extension movements of the SAZ. Vestiges of these three segmentation mechanisms can be found in almost all extant arthropods.

I raise the intriguing possibility that originally, the distinction between the second segmentation mechanism (segmentation within a pre-existing developmental field) and the third mechanism (segmentation from an SAZ), was a distinction between embryonic and post-embryonic segmentation. In a previous paper [86], I made the implicit assumption that the common ancestor of all arthropods was a direct developer forming all segments during embryogenesis, and that indirect development evolved convergently in different lineages. This was based mostly on lack of evidence for indirect development in the fossil record. Subsequently, Wolfe [87] argued that metamorphosis was ancestral, based on phylogenetic considerations. With the new data available, I suggest a revised and intermediate solution. The last arthropod common ancestor was hemianamorphic (i.e. some segments were formed in embryogenesis and some post-embryonically). The anamorphically forming segments were generated via an SAZ in a gradual process, with new segments probably appearing during the moulting process. Indeed, the SAZ may have evolved as a developmental mechanism for post-embryonic segment generation. Since the closest sister groups to arthropods (onychophorans and tardigrades) both generate all segments during embryonic development, we cannot say for certain when post-embryonic segmentation, and by extension the SAZ, evolved. However, the fact that we find post-embryonic segment addition in a radiodont provides a possible phylogenetic bracketing [80].

The extent of the segments formed in a pre-existing field (hereafter PEF segments) varies in different arthropods. This field still exists as a clear and distinct field in arachnids and in insects. In insects, it comprises six segments (gnathal + thoracic). In arachnids, it comprises four segments (walking limbs). In crustaceans with a nauplius larva, it may have been lost, with only one post-PGS segment (the mandibular segment) patterned not via the SAZ. Nonetheless, in some crustaceans, the first few post-naupliar segments appear simultaneously, which may be a vestige of this mechanism. In myriapods, the pre-existing field is not as distinct, but the dynamics of segmentation suggests that the SAZ only starts generating segments from the first or second trunk segment, indicating that at least the embryonic gnathal segments are within this field.

Most of the diversity of developmental tagmosis modes in different arthropods can be seen as stemming from variation in three parameters: (i) the number of segments patterned under each of the mechanisms, (ii) the point in development at which the germ band condenses and gastrulation takes place, and (iii) the stage of development at which the embryo hatches. The latter two are based on heterochronic shifts, which can occur fairly rapidly and easily from an evolutionary point of view. The former tends to be conserved at high taxonomic levels, although departures from the taxon-typical mode can be found. The diversity of arthropods is such that there are many examples of unusual development and tagmosis. The strangeness of these examples should not distract us from identifying the deeply rooted ancestral pattern.

## 6. Implications of the model

### (a) Short-germ versus long-germ development

For nearly a century, the literature on insect and arthropod development has made the distinction between short-germ development and long-germ development [88–90]. The difference lies in the extent of the embryo that is patterned before gastrulation, or germ-band condensation. In long-germ development, the entire embryonic axis is patterned prior to gastrulation, and in short-germ development only a small part of the axis is (usually only the head). Intermediate-germ is used for cases where the head and thorax are patterned prior to gastrulation. Short- and long-germ development are often also used to make a distinction between simultaneous and sequential segmentation, although this was not the original meaning of the terms.

The proposed model for the evolution and development of tagmata requires a reframing of long- and short-germ development in the context of the different embryonic tagmata. Two separate modes of segmentation exist in almost all arthropods, except for in the most extreme cases of long-germ simultaneous segmentation such as *Drosophila*. The main difference between the modes is in the heterochronic shift of gastrulation and germ-band condensation relative to the segmentation process.

### (b) The mandibulate head

The head of mandibulate arthropods (myriapods, crustaceans and insects) incorporates segments from two embryonic tagmata: the PGS and the PEF segments [91,92]. The fossil record shows that the ancestral head in crown group arthropods was composed of only the PGS [26]. The evolution of the mandibulate head initially involved the fusion of the entire PEF tagma with the PGS to give rise to the six-segment head (although stem mandibulate fossils suggest this may have originally been only two PEF segments and a five-segmented head [24]). This is the case in extant myriapods and may be the ancestral mode in crustaceans (but see discussion of head larvae in §6d).

### (c) The insect thorax

Insects are the largest class within arthropods and the most conservative in terms of their overall body plan (despite being extremely variable in modifications of this body plan). Perhaps one of the sources of the insects’ success is the tightly integrated thorax, which specializes in locomotory function, and has no direct equivalent in any other arthropods class. The uniqueness of the insect thorax extends to its developmental origin. There is no other case where the PEF tagma subdivides into two functional adult tagmata. In myriapods and in non-hexapod crustaceans, the PEF is small—probably only three segments—and is entirely incorporated into the mandibulate head. In insects, this tagma encompasses six segments, half of which are incorporated into the head and half of which form the thorax. This realization puts the evolution of the insect body plan in a new light and suggests additional avenues for evo-devo research, while also predicting potential transitional body plans that might be found in the fossil record.

### (d) The issue of head larvae

The term ‘head larva’ is often used to describe larval forms that comprise only 3−4 segments, essentially the PGS and possibly an additional gnathal segment. The best-known head larvae are the nauplius larvae common in many crustaceans (and possibly plesiomorphic for Pancrustacea), but head larvae are also found in some pycnogonids and in horseshoe crabs. Because of their highly derived post-embryonic development mode, it is difficult to incorporate these forms into the current model. However, looking at the cases where there is an embryonic nauplius (egg-nauplius) helps clarify the situation. As mentioned above, many crustaceans exhibit a slightly different mode of segmentation in the anterior few gnathal/trunk segments, suggesting that there is an anterior tagma that is distinct from the SAZ-driven sequentially segmenting posterior tagma. The evolution of head larvae involved an extreme heterochronic shift in the time of hatching relative to segment formation. The postponement of segmentation to the post-embryonic period obscures the different segmentation modes, and they may even be lost in some lineages.

### (e) Homology of tagmata

The view articulated by Fusco & Minelli [1] is that tagmata cannot be homologized across arthropod classes. I argue that if all PEF tagmata are derived from an ancestral embryonic tagma that was defined embryonically in a PEF, they should be seen as homologous. Similarly, all SAZ-derived tagmata are homologous (it is already generally accepted that the PGS are homologous across arthropods). However, although similar terms are used in different lineages (cephalothorax, trunk), there is no way to unambiguously homologize specific segments or structures within a given tagma across arthropod phylogeny. When comparing species with the same number of segments in a specific tagma, it may be possible to equate a segment in a given position with a segment in the same position in a different species, but this should be done with caution and with consideration of the evolutionary history of the species being compared.

### (f) The role of Hox genes in segment identity

The prevailing paradigm is that Hox genes are the earliest determinants of segment identity in arthropods. Specific Hox genes confer tagma fate (e.g. *Antennapedia* is responsible for thoracic fate). Data on the development of numerous arthropods reviewed above indicate that the difference between the thorax and abdomen in insects or between the prosoma and opisthosoma in arachnids is determined very early in development, before the segments are visible morphologically, even before the segmentation cascade is completed, and indeed before Hox gene expression begins in the relevant regions.

There is no doubt that Hox genes have a role in specific segment identity and this is supported by an enormous body of evidence, not only from *Drosophila melanogaster,* but from many other species [93]. Nonetheless, we must reconsider their role in determining higher level identity (i.e. the distinction between segments belonging to different tagmata), based on the observation that these differences stem from very early embryonic differences.

I suggest a two-phase model for segment identity determination. The first phase defines tagma identity based on embryonic differences in the segmentation process of segments in the different tagmata. The genes responsible for these differences are currently unknown. The second phase defines the morphological differences between the segments and is responsible for the development of segment- and tagma-specific modifications. This phase is largely driven by Hox genes. The level of integration and mutual regulation and feedback between these two processes is likewise unknown and can form the basis for a fruitful research agenda.

## 7. Conclusions

The evolution and development of tagmosis have been discussed intermittently in the arthropod literature for decades, but there has never been an attempt to offer a synthetic model for tagmosis. Most of the data presented in this paper have been known for many years. Crucial pieces of the puzzle were provided by the more recent study of segmentation in hemimetabolous insects within the context of tagma identity. I have attempted to provide a model of tagmosis that takes into account morphology, embryonic and post-embryonic segmentation, developmental dynamics, phylogeny and data from the fossil record. I have attempted to identify the core conserved elements of the developmental basis of tagmosis, while disregarding the many lineage-specific variations in tagmosis that have mostly led to confusion in the field. The synthetic model I present provides a relatively simple explanation for the observed patterns of extant and fossil arthropod body plans and also opens a slew of new questions that can—and should—be studied experimentally in a range of arthropod species.

## Data Availability

Supplementary material is available online [94].
